# Readjustment of abdominal computed tomography protocols in a university hospital: impact on radiation dose*

**DOI:** 10.1590/0100-3984.2014.0054

**Published:** 2015

**Authors:** Ricardo Francisco Tavares Romano, Priscila Silveira Salvadori, Lucas Rios Torres, Elisa Almeida Sathler Bretas, Daniel Bekhor, Rogério Pedreschi Caldana, Regina Bitelli Medeiros, Giuseppe D’Ippolito

**Affiliations:** 1 Collaborating Physicians, Department of Imaging Diagnosis at Escola Paulista de Medicina – Universidade Federal de São Paulo (EPM-Unifesp), São Paulo, SP, Brazil.; 2 Masters, Physicians Assistants, Department of Imaging Diagnosis at Escola Paulista de Medicina – Universidade Federal de São Paulo (EPM-Unifesp), São Paulo, SP, Brazil.; 3 MD, Fellow, Department of Imaging Diagnosis at Escola Paulista de Medicina – Universidade Federal de São Paulo (EPM-Unifesp), São Paulo, SP, Brazil.; 4 PhD, MD, Radiologist, Fleury Medicina e Saúde, São Paulo, SP, Brazil.; 5 Affiliate Professor, Department of Imaging Diagnosis at Escola Paulista de Medicina – Universidade Federal de São Paulo (EPM-Unifesp), São Paulo, SP, Brazil.; 6 Private Docent, Associate Professor, Department of Imaging Diagnosis at Escola Paulista de Medicina – Universidade Federal de São Paulo (EPM-Unifesp), São Paulo, SP, Brazil.

**Keywords:** Computed tomography, Abdomen, Contrast media, Radiation dose, Radiation protection

## Abstract

**Objective:**

To assess the reduction of estimated radiation dose in abdominal computed tomography following the implementation of new scan protocols on the basis of clinical suspicion and of adjusted images acquisition parameters.

**Materials and Methods:**

Retrospective and prospective review of reports on radiation dose from abdominal CT scans performed three months before (group A – 551 studies) and three months after (group B – 788 studies) implementation of new scan protocols proposed as a function of clinical indications. Also, the images acquisition parameters were adjusted to reduce the radiation dose at each scan phase. The groups were compared for mean number of acquisition phases, mean CTDI_vol_ per phase, mean DLP per phase, and mean DLP per scan.

**Results:**

A significant reduction was observed for group B as regards all the analyzed aspects, as follows: 33.9%, 25.0%, 27.0% and 52.5%, respectively for number of acquisition phases, CTDI_vol_ per phase, DLP per phase and DLP per scan (*p* < 0.001).

**Conclusion:**

The rational use of abdominal computed tomography scan phases based on the clinical suspicion in conjunction with the adjusted images acquisition parameters allows for a 50% reduction in the radiation dose from abdominal computed tomography scans.

## Abstract (pt)

**Objetivo:**

Quantificar a redução da dose estimada de radiação em exames de tomografia computadorizada de abdome após a implementação de novos protocolos dirigidos para a suspeita clínica e ajuste nos parâmetros técnicos de aquisição.

**Materiais e Métodos:**

Foram avaliados, de forma retrospectiva e prospectiva, os relatórios de dose de exames de tomografia computadorizada de abdome realizados três meses antes (grupo A – 511 exames) e três meses após (grupo B – 788 exames) a implementação de novos protocolos de exame propostos em função das indicações clínicas. Contemporaneamente, os parâmetros de aquisição das imagens foram ajustados de modo a reduzir a exposição em cada fase do exame. Os grupos foram comparados quanto ao número médio de fases de aquisição, valores de CTDI_vol_ por fase, DLP por fase e DLP por exame.

**Resultados:**

O grupo B apresentou redução significativa em todos os aspectos analisados: número de fases, CTDI_vol_ por fase, DLP por fase e DLP por exame mostraram reduções de 33,9%, 25,0%, 27,0% e 52,5%, respectivamente (*p* < 0,001).

**Conclusão:**

O uso racional das fases de aquisição dirigidas para a suspeita clínica, em conjunto com adequação nos aspectos técnicos, permite reduzir pela metade a dose de radiação por exame de tomografia computadorizada de abdome.

## INTRODUCTION

Computed tomography (CT) has brought invaluable improvements to clinical practice since its inception, replacing other diagnostic methods due to its swiftness, efficiency and accuracy. However, the increasing number of indications and wide availability of the method have led to a significant increase in the number of CT scans, and consequently, in the radiation dose to which patients are exposed^(1)^.

According to data from the National Council on Radiation Protection and Measurements, one estimates that radiation dose has almost doubled since 1980, mainly on account of imaging exams, which contributed for a seven-fold increase in the population dose, overcoming the exposure caused by environmental factors^(1)^. Over the same period, the number of CT scans increased 20 times, from 3 million to 60 million scans per year, and nowadays it is responsible for a quarter of the population exposure to radiation^(2)^.

With a view on such a context, an increasing willingness is observed among physicians and regulatory authorities^(2)^ to find means to reduce radiation exposure of patients during CT scans. Strategies such as establishment of clinical criteria for the realization of the scans and development of clinical investigation algorithms, reduction of the number of images acquisition phases^(3)^, reduction of the area to be scanned^(4)^ and rational utilization of the images acquisition technical parameters^(5)^ have been successfully adopted at institutions all over the world^(6)^, with the objective of controlling the radiation exposure.

Recent studies demonstrated that it is possible to decrease the number of acquisition phases in accordance with the clinical indication, maintaining the diagnostic effectiveness unchanged^(7,8)^. Such a fact motivated a complete review of abdominal computed tomography protocols at the author's institution as well as the deployment of a training program for all professionals involved in the performance of scans, including radiologists, residents, technicians and nursing personnel.

With the development of such new protocols for CT scan directed to the clinical suspicion, the rationalization of technical aspects related to image acquisition, and the training of the service professionals, the present study authors aimed at quantitatively evaluating dose reduction at abdominal CT that such strategies determined in the scans performed at the institution.

## MATERIALS AND METHODS

A retrospective, prospective, observational study was undertaken, evaluating the dose reports from the abdominal CT scans performed at the authors' university hospital over two different periods, as follows: a) during three months prior to the implementation of the new protocols (September, October and November of 2012); b) during three months after the implementation of such protocols (March, April and May of 2013). The study was approved by the Committee for Ethics in Research of the institution (register No. 296.803), with the need for a term of free and informed consent being waived by the Committee.

Inclusion criteria were abdominal and pelvic CT scans of patients aged above 18, performed in the authors' institution during the above mentioned periods. Exclusion criteria were the following: a) patients submitted to CT scan in other body segments concomitantly to the abdomen and pelvis scan, due to the difficulty in establishing the limits of image acquisition and respective radiation dose; b) incomplete exams or those with technical errors.

A total of 1299 CT studies were evaluated, 511 performed before the scan protocol review (group A) and 788 performed after the implementation of the new scan protocols (group B).

All scans were performed in Brilliance 64^®^ 64-channel scanner (Philips Medical Systems; Cleveland, USA) utilizing built-in automatic dose modulation supplied by the manufacturer (Z-DOM^®^).

Whenever the use of intravenous iodinated contrast was indicated, the injection was made according to parameters on Table 1. In those cases where the angiographic or arterial phases were indicated, an automatic bolus-tracking system was utilized (Bolus Tracking - ScanTools Pro^®^).

**Table 1:** Image acquisition parameters.

Contrast	
Abdomen and pelvis	1.5 mL/kg (350 mOsm/L) or 2.0 mL/kg (300 mOsm/L) up to 150 mL
	3 mL/s (5 mL/s if arterial phase)
Angiography	1.0 mL/kg (350 mOsm/L) up to 100 mL; 5 mL/s
Aquisition phases	
Pre-contrast	—
Arterial	20 s after 100 UH threshold in the aorta or 40 s after contrast injection
Portal	20 s after arterial phase or 75 s after contrast injection
Equilibrium	2 min after portal phase or 3 min after contrast injection
Delayed	7 to 10 min after portal phase
Extent	
Abdomen + pelvis	Diaphragmatic dome to pubic symphysis
Abdomen	Diaphragmatic dome to iliac crest

Initially, a training program was provided for the technicians involved in the operation of the CT scanner, nursing team, physicians from the diagnostic radiology residence program and from the abdominal radiology sector, approaching the objectives to be achieved and the changes proposed for that purpose. Such a training program was carried out on an in-class basis, with audiovisual presentations and posters with the new guidelines.

Once this stage was completed, a survey on the most common indications for abdominal CT scans at the authors' institution was carried out in order to determine which ones should have the scan protocols reviewed. Once the necessary acquisition phases for the selected clinical indications were determined with basis on the authors' previous experience^(7,8)^, a set of scan protocols was designed in order to cover the clinical indications in a simplified way, utilizing guidelines and recommendations widely published in the literature^(4)^.

The comparison of radiation doses was made by means of the CT dose index values (CTDI*vol*) and the dose length product (DLP) from each scan in groups A and B, respectively. The DLP represents the radiation dose of one CT section multiplied by the study length and it is measured in mGy/cm. The effective radiation dose (that estimates the total risk for stochastic effects caused by an irradiated organ exposure) can be calculated by multiplying the DLP by a correction factor according to the studied anatomic region^(8)^. The correction factor is utilized for calculation of the effective dose (expressed in mSv) and, at abdominal CT, it ranges from 0.015 to 0.018^(9)^. For the purposes of calculation, a correction factor of 0.015 mSv/mGy*cm was utilized in the present study.

The result obtained from such a calculation is not the exact value of the estimated radiation, but it can be utilized as a reference value at a given CT service. Considering that there is great practical difficulty in measuring the exact dose per patient, because of the many variables inherent to the patient involved in the calculation (for example: body mass index, abdominal circumference, irradiated organ) and inherent to technical factors (for example: kV, mAs, pitch)^(9)^.

### Acquisition protocols

Based on evidences reported in the literature, scan phases sets were established according to clinical suspicion. In this process, the authors made an effort towards maintaining the lowest possible viable number of protocols and organizing them in such a way to simplify their prescription and their adoption by the technicians in charge of the scans (Table 2).

**Table 2:** Scan protocols.

		Unenhanced	Arterial	Portal	Equilibrium	Delayed
1	Unenhanced	Abdomen + pelvis				
2	Contrast-enhanced - single phase			Abdomen + pelvis		
3	Hypovascular	Abdomen + pelvis		Abdomen + pelvis		
4	Hypervascular		Abdomen	Abdomen + pelvis		
5	Hepatic nodule	Abdomen	Abdomen	Abdomen + pelvis	Abdomen	
6	Urotomography	Abdomen + pelvis	Abdomen	Abdomen + pelvis		Abdomen + pelvis
7	Trauma	Abdomen + pelvis	Abdomen + pelvis (angiographic arterial phase)	Abdomen + pelvis		Abdomen + pelvis
8	CT angiography	Abdomen + pelvis	Abdomen + pelvis (angiographic arterial phase)	Abdomen + pelvis		
9	Adrenal nodule	Abdomen + pelvis		Abdomen (if necessary)		Abdomen 15 min (if necessary)

1. "Unenhanced scan" protocol: was adopted for investigation of renal lithiasis, appendicitis and acute diverticulitis as well as for those patients for whom the utilization of iodinated contrast is contraindicated^10)^.
2. "Contrast-enhanced single phase" protocol: such protocol was adopted for the restaging of hypovascular tumor^(11)^, acute pancreatitis^(12)^, complicated pyelonephritis^(13)^, and investigation of intracavitary collections^(7,8)^.
3. "Hypovascular" protocol: utilized in cases where it is necessary to evaluate the enhancement of abdominal structures, as in cases of acute abdomen with unknown causes, abdominopelvic masses and follow-up of hematomas, as well as in initial staging of hypovascular tumors (for example: rectal carcinoma)^(11)^.
4. "Hypervascular" protocol: performed to investigate hypervascular neoplastic lesions (for example: investigation of melanoma metastases, carcinoid tumors, etc.), being adopted in the investigation, staging and restaging of such neoplasms^(14)^.
5. "Hepatic nodule" protocol: for characterization of focal hepatic lesions^(15)^ and investigation of hepatocellular carcinoma^(16)^.
6. "Urotomography" protocol: utilized in the suspicion of urinary tract lesions^(17)^, and more specifically in the investigation of causes of hematuria.
7. "Trauma" protocol: indicated in the evaluation of patients presenting with abdominal trauma. In such a protocol a portal acquisition is performed for characterization of lesions in solid organs and abdominal vessels, and a delayed phase, for evaluation of the urinary system^(18)^. A further arterial phase depends upon the suspicion of high energy lesion or pelvic trauma^(19)^.
8. "Abdominal CT angiography" protocol: it is utilized in the suspicion of aortic and splanchnic aneurysms and other vascular anomalies, follow-up of endoprostheses^(20)^, as well as in cases of vascular acute abdomen^(21)^.
9. "Adrenal" protocol: the characterization of adrenal nodule with respect to its histological behavior depends particularly on its attenuation before the contrast injection as well as on the enhancement curve behavior after contrast injection^(22)^. In this context, the scan is interrupted after the unenhanced phase in cases where the nodule density is < 10 UH; otherwise, the scan is completed with the contrast-enhanced phases^(23)^.

The collection of the estimated dose values per scan was performed by means of a standardized table generated by the CT scanner, attached to the DICOM images. The statistical analysis was performed by means of the SPSS 20 program (IBM; USA), utilizing the Student *t* test for independent variables, considering the values corresponding to number of phases, CTDI_vol_ per phase, and DLP per scan in the groups A and B. The confidence interval was calculated for two standard deviations (SD) and values of *p* < 0.05 were considered to be statistically significant.

## RESULTS

Groups A and B were initially analyzed with respect to their composition in terms of gender and age. The ages presented similar distribution, with no statistically significant difference (*p* = 0.024). The distribution by gender was also similar in both groups.

The data collected before and after the changes in the acquisition protocol (groups A and B) as regards number of phases per scan, CTDI_vol_ per phase, DLP per phase, and DLP per scan are shown on Table 3.

**Table 3:** Number of phases and mean radiation dose index per phase and per scan, in groups A and B.

	Group A (*n* = 511)		Group B (*n* = 788)				
	Mean	SD	Mean	SD	Difference	CI 95%	*p* (< 0.05)
Number of phases	2.68	1.14	1.77	1.09	0.91 (33.9%)	0.78-1.03	< 0.001
CTDI_vol_ per phase	16.39	5.39	12.28	4.21	4.11 (25.0%)	3.56-4.67	< 0.001
DLP per phase	814.37	300.41	594.88	2.25	219.48 (27.0%)	188.98-249.99	< 0.001
DLP per scan	2222.32	1198.87	1053.97	761.18	1168.35 (52.5%)	1051.42-1285.28	< 0.001

SD, standard deviation; CI 95%, confidence interval 95%; *p* < 0.05 is considered as statistically significant.

### Number of acquisition phases

Among the evaluated individual parameters, the number of acquisition phases performed per scan presented the greatest decrease; the mean number of phases per scan in group A was 2.68 (SD = 1.14), and in group B it was 1.77 (SD = 1.09). In other words, there was an average decrease of 33% in the number of performed phases after the review of the protocols.

As the distribution of number of phases in both groups is analyzed, one observes that in group A approximately 30% of the scans were performed with one or two phases, differently from group B, where approximately 80% of the scans were performed with one or two phases (Table 4).

**Table 4:** Change in the number of acquisitions as groups A and B are compared.

Number of aquisitions	Group A	Group B
One phase	147 (28.8%)	453 (57.5%)
Two phases	0 (0%)	182 (23.1%)
Three phases	233 (45.6%)	31 (3.9%)
Four phases	131 (25.6%)	122 (15.5%)

### CTDI_vol_ and DLP per phase

In the present study, the adjustment of the acquisition parameters, such as smaller acquisition extent, mAs reduction and the routine use of the automatic dose modulation program, determined a significant reduction (*p* < 0.001) of the CTDI_vol_ per phase and DLP per phase, respectively of 25% and 27% per acquisition.

### DLP per scan

In the present study, a significant reduction (*p* < 0.001) of 52.5% was observed (from 2222.32 mGy*cm to 1053.97 mGy*cm, between group A and group B), which is equivalent to a mean effective dose reduction per scan of 33.33 mSv to 15.98 mSv, as the conversion factor of 0.015 mSv/mGy*cm is applied.

## DISCUSSION

Approaches such as reduction of X-rays generation^(24)^, limitation of the area to be scanned^(4)^, improved reconstruction algorithms^(25)^ and reduction in the number of acquisitions^(3,8)^ have been the subject of a great number of studies over the past decade^(6-8,14-26)^ and have allowed the performance of abdominal CT scans with effective dose values similar to those of specialized radiological exams^(26)^.

As the abdominal CT protocol rationalization program is applied in the authors' institution, a decrease of approximately 50% of the mean radiation dose per scan was observed. Among the factors determining such decrease, one should highlight that the mean reduction of approximately 33% in the number of phases and the reduction of CTDI_vol_ and DLP in approximately 25% were the main contributors to the dose reduction.

Most of the major abdominal syndromes have a presentation that allows for the elimination of some acquisition phase, considering a complete four-phase protocol (non-contrast-enhanced, arterial, portal and equilibrium phases)^(7,8)^. Thus, a careful evaluation of the clinical suspicion allows for the reduction of the number of phases to a justifiable minimum^(3)^.

During the period of evaluation and validation of the new protocols with the group of professionals involved in the realization of CT scans, one difficulty stood out: the resistance imposed from some of the most experienced radiologists in modifying established practices. As already demonstrated in the literature, the elimination of acquisition phases does not necessarily imply reduction in accuracy for many clinical situations frequently faced in a CT service. However the habit of utilizing all available phases actually originated from the education of many professionals, in a time when a supposedly more thorough examination was recommended, even in cases where such a strategy did not result in a significantly increased diagnostic effectiveness of the method^(3,7,8)^.

At a similar level, the improvement of the X-ray generation parameters provided the authors with a distinctive approach for dose reduction. Without compromising the diagnostic quality of the images, as previously demonstrated^(8)^, such an improvement allowed for 25% estimated dose reduction in all scans, including the four phases and single phase scans. The resulting mean CTDI_vol_ per phase is quite close to that observed in an experimental study on dose reduction^(27)^ and is considered to be appropriate for accreditation by the radiological societies which adopt such parameter^(28)^.

Besides the reduction of radiation exposure, the optimization of the protocols brought additional benefits such as reduction in the time required to perform the scans. Previously to the protocols review, 71.2% (364/511 scans) of the patients remained for at least 7 minutes inside the CT scanner. After implementation of the new protocols, only 15.5% of the patients (122/768 scans) were submitted to the equilibrium phase. Considering the number of abdominal scans in the hospital environment, and that both the patient and the apparatus have to wait for approximately three minutes for the start of the equilibrium phase, the elimination of such a phase saved time enough to allow for the performance of more scans per day, besides a shorter patient stay inside the scanner.

Another secondary outcome was the better utilization of the CT scanner and the data storage resources. As the present study sample is considered, 1370 acquisitions were performed in 511 scans before the protocols review (on average, 2.68 acquisitions per scan) and 1398 acquisitions in 788 scans after the protocols review (on average, 1.77 acquisitions per scan). As the number of phases is reduced, the wear of the apparatus also decreases, allowing for longer service life of the X-ray tube, and reduction of the number of images to be processed and stored.

One of the precautions that must be taken into consideration in the review of scans protocol is related to the maintenance of the diagnostic efficiency and effectiveness of the method. The efficiency can be measured by the proportion of recalls required to supplement scans initially considered to be insufficient. The effectiveness can be indirectly evaluated by calculation of added value for each additional acquisition phase. In the present study, there was constant control on the number of recalls and their respective reasons. Over the study period, the monthly average number of recalls remained practically unchanged, with nine supplementary scans in the trimester for each studied group, corresponding to, respectively, 1.7% (group A) and 1.1% (group B) of the scans. Such recalls occurred mainly because of the utilization of inappropriate scan protocols in patients whose complete clinical history was not available at their initial evaluation. In spite of the recall being uncomfortable for the patient and representing extra radiation exposure, the present study authors believe that the benefit from the optimized protocols are maintained, both in individual terms, as the patient will be exposed to a lower effective radiation dose, as in population terms, with lower mean radiation doses.

As regards the effectiveness of the protocols adopted in the present study, it was possible to demonstrate, in studies previously developed by the same authors^(7,8)^, as well as studies developed in other research centers^(3,11,12,27)^, that scan protocols directed to clinical suspicion do not significantly affect the diagnostic accuracy when properly implemented. For this reason, the evaluation of the CT accuracy after implementation of the new protocols in the daily routine was not included in the scope of the present study that was focused on the radiation dose measurement.

The authors identify some limitation in the present investigation. The first one refers to the fact that when quantifying the reduction of radiation exposure, the authors utilized the dose report generated by the scanner itself, without taking into consideration the patients' weight and body constitution - determining factors in the correct effective dose estimation. However, in both groups, the authors studied all adult patients from both genders and with similar distribution, who had been routinely admitted into the service, which allowed for a reliable comparison, as variations in weight and body constitution disperse in the sampling. A similar limitation was observed in other multicentric study^(29)^. The second limitation lies on the fact that additionally to the changes implemented during the present study, there is an opportunity for further reduction of radiation dose during CT scans by means of other techniques such as the split-bolus contrast injection, where one image acquisition corresponds to multiple phases of concomitant enhancement. Finally, the authors did not utilize iterative reconstruction algorithms (iDose^®^; Philips Medical Systems), which would allow for even lower radiation levels^(25)^, since such a resource was not available in the equipment utilized in the present study. The authors believe that the utilization of such techniques would result in even more significant results.

## CONCLUSION

The application of concepts such as review of imaging parameters and rational utilization of multiple acquisition phases in the authors' institution allowed for the reduction by half of the mean dose index per abdominal CT scan. In the current scenario where the utilization of CT in the clinical practice is increasingly frequent, it is of utmost importance that the scans be performed in a way to minimize the exposure of patients to radiation. The radiologist is responsible for constantly reviewing and updating such protocols.
